# The Impact of Age on the Physiological Assessment of Borderline Coronary Stenoses

**DOI:** 10.3390/medicina59101863

**Published:** 2023-10-19

**Authors:** Wojciech Zasada, Barbara Zdzierak, Tomasz Rakowski, Beata Bobrowska, Agata Krawczyk-Ożóg, Sławomir Surowiec, Stanisław Bartuś, Andrzej Surdacki, Artur Dziewierz

**Affiliations:** 1 Clinical Department of Cardiology and Cardiovascular Interventions, University Hospital, 30-688 Krakow, Poland; zasada.wojciech@gmail.com (W.Z.); mcrakows@cyf-kr.edu.pl (T.R.);; 2 KCRI, 30-347 Krakow, Poland; 3 2nd Department of Cardiology, Institute of Cardiology, Jagiellonian University Medical College, 30-688 Krakow, Poland; 4 Department of Anatomy, HEART-Heart Embryology and Anatomy Research Team, Jagiellonian University Medical College, 31-034 Krakow, Poland

**Keywords:** age, borderline stenoses, fractional flow reserve, instantaneous wave-free ratio, physiological assessment, resting full-cycle ratio

## Abstract

*Background and Objectives*: Coronary angiography is the gold standard for diagnosing coronary artery disease (CAD). In the case of borderline changes, patients require further diagnosis through ischemia assessment via one of the recommended methods of invasive evaluation. This study aimed to assess whether clinical factors influence the risk of a positive result in invasive myocardial ischemia assessment and if these potential factors change with the patient’s age and the consistency of ischemia assessment. *Materials and Methods*: Data were collected retrospectively on all consecutive patients hospitalized in the University Hospital in Krakow between 2020 and 2021, on whom physiological assessments of coronary circulation were performed. Patients were divided into two groups: patients aged 60 or younger and patients older than 60. *Results*: Despite the older patients having more risk factors for CAD, their physiological assessment results of borderline lesions were similar to those of the younger patients. Positive fractional flow reserve (FFR) assessments were obtained from almost 50% of vessels. In the younger patients, cigarette use and type 2 diabetes mellitus increased the risk of a positive FFR result by 3.5 and 2.5 times, respectively. In the older patients, male gender and peripheral vascular disease significantly increased the risk of a positive FFR by 2.5 and 2 times, respectively. *Conclusions*: Clinical characteristics of patients undergoing physiological assessment of borderline coronary stenosis varied significantly by age. Refining the definition of borderline lesions to include age, gender, and other factors may improve the identification of patients who would benefit from physiological assessment and coronary revascularization.

## 1. Introduction

Coronary artery disease (CAD) is one of the most prevalent heart diseases among adult patients [1]. Atherosclerotic changes in coronary arteries, which limit blood flow to the myocardium, are the most common cause of myocardial ischemia [2]. The gold standard for diagnosing CAD is coronary angiography, which visualizes the presence and localization of changes responsible for myocardial ischemia. This procedure often involves simultaneous coronary revascularization. CAD can be ruled out if angiography reveals no abnormalities. However, borderline changes, defined as 50–90% narrowing of the vessel lumen, may be present during the examination, rendering angiography insufficient for determining if such a change causes significant myocardial ischemia. In these cases, patients require further diagnosis through ischemia assessment, with one of the recommended methods being invasive assessment (class IA recommendation [3]). Currently, the gold standard for this assessment is measuring the pressure gradient before and after stenosis during induced hyperemia, which is typically achieved by administering adenosine-the fractional flow reserve (FFR) test. Invasive myocardial ischemia assessment includes several hyperemia-independent methods, such as the instantaneous wave-free ratio (iFR) and resting full-cycle ratio (RFR).

Certain well-established factors significantly affect the risk of developing CAD, including diabetes mellitus, smoking, arterial hypertension, lipid metabolism disorders, and genetic factors [4]. Furthermore, it is known that, depending on a patient’s age, different factors may play a more prominent role in the development of CAD. Thus, we aimed to assess whether clinical factors influence the risk of a positive result in invasive myocardial ischemia assessment, if these potential factors change with the patient’s age, and if myocardial ischemia assessment is consistent when using the available methods (FFR, iFR/RFR).

## 2. Methods

Data were collected retrospectively on all consecutive patients hospitalized at the Clinical Department of Cardiology and Cardiovascular Interventions of the University Hospital in Krakow between 2020 and 2021, in whom physiological assessment of the coronary circulation was performed. A database containing information about the basic demographic characteristics of treated patients, the presence of risk factors for ischemic heart disease, and their history of chronic diseases and treatments was prepared. In addition, selected data were collected from each patient’s physical examination at admission (e.g., height, weight, and left ventricular ejection fraction) and the results of the laboratory tests performed when the patient was admitted to the department. Data regarding the results of coronary angiography and the results of the physiological assessment of the coronary circulation were also collected. The database prepared in this way included information about 318 consecutive patients who qualified for physiological assessment of the coronary circulation during invasive diagnosis of ischemic heart disease. At the same time, data regarding the results of the physiological assessment of 417 vessels were collected. For further analysis, the examined patients were divided into two groups: The first group comprised patients aged 60 years or younger (‘Younger group’). The second group consisted of patients older than 60 (‘Older group’). The exact cutoff point for age was used in previous studies [5] due in part to reports of significant discrepancies in coronary blood flow during hyperemia [6] and myocardial perfusion reserve [7] after the age of 60.

Coronary angiography was performed on each eligible patient by an experienced operator in accordance with the standard for this procedure adopted in the center. The operator decided on the choice of vascular access—in most cases, the access point was radial or, less frequently, femoral. Angiography was performed using 6F diagnostic catheters, and the curves were individually selected by the operator to best suit the anatomy of the examined patient. The invasive physiological assessment procedure was performed each time according to the operator’s decision. In each situation when, in the operator’s opinion, the atherosclerotic lesions in the coronary arteries shown on angiography were borderline, he had the opportunity to perform a physiological assessment of the coronary circulation, as the mismatch between coronary stenosis’s angiographic and hemodynamic severity is frequent [8]. Borderline lesions were defined angiographically as lesions narrowing the vessel lumen in the range of 50–90% diameter stenosis as visually assessed by the operator. The operator selected the optimal vascular access, catheter type, method of inducing hyperemia, and pressure wire type, all of which then determined the non-hyperemic assessment method.

A physiological assessment of the coronary circulation was performed immediately after coronary angiography. Invasive physiological assessment of the coronary circulation was performed using a diagnostic catheter or a guide catheter at the operator’s discretion. The assessment protocol was first intended to complete a non-hyperemic assessment. Depending on the choice of the system made by the operator, the non-hyperemic assessment was performed as RFR or iFR. Based on the dedicated software, RFR was performed using Abbott’s PressureWire™ guide wire and calculated from the lowest value of Pd/Pa over the entire cardiac cycle. Pd is the ‘distal pressure’—pressure measured using a sensor placed on the guidewire distal to the stenosis being assessed. In turn, Pa is the ‘proximal pressure’—pressure measured in the aorta using a sensor connected to the catheter. iFR assessment was performed using a Philips pressure guide wire. The iFR modality measured pressure during the wave-free period of the cardiac cycle, ranging from mid- to late diastole, when resistance is naturally constant. The result of this measurement is, similarly to the RFR, the Pd to Pa ratio. Typically, iFR was measured during the subsequent five heart cycles. Regardless of the choice of non-hyperemic assessment, both RFR and iFR were performed at least three times. If any of the three measurements were unreliable in the investigator’s opinion, e.g., due to the appearance of artifacts, patient movement, irregular heartbeat, deep breathing, or coughing, the measurement was repeated. Finally, once three correct measurements performed correctly were obtained by the researcher, the final result was determined from the average value of the results obtained.

After performing the non-hyperemic assessment, the operator performed the FFR. The FFR was measured using the same pressure wire used for non-hyperemic assessment. The method of obtaining hyperemia was selected each time by the operator. Hyperemia was most often achieved by intracoronary boluses of adenosine at a dose ranging from 100 µg to 400 µg. If, in the operator’s opinion, the obtained FFR result was not reliable, for example, due to failure to obtain hyperemia, incorrect position of the pressure wire, or the appearance of the artifacts, the measurement was repeated until the correct recording of pressure curves and a reliable FFR result were achieved. The procedure of physiological assessment of the coronary circulation was performed for all lesions visible on angiography that the operator considered borderline. After completing the physiological assessment of the coronary circulation, further therapeutic decisions were left to the operator’s discretion.

Because both methods are considered equivalent [9], the results were combined and treated as non-hyperemic assessment outcomes, regardless of the method used. The values of ≤0.80 for FFR and ≤0.89 for iFR/RFR were considered positive for ischemia. The results of the FFR assessment, as well as the non-hyperemic assessment, were analyzed both as a binary variable—positive/negative result for myocardial ischemia,—and as a continuous variable. The study groups’ demographic characteristics and medical history were analyzed, and the analysis was followed by comparing the hyperemic and non-hyperemic evaluation results. This comparison was presented for all vessels analyzed in individual study groups and separately for the left anterior descending artery (LAD) and non-LAD. In the non-LAD group, the following vessels were assessed: diagonal branches, the circumflex artery, marginal branches, and the right coronary artery. Lesions within the left main coronary artery, as well as in the saphenous vein or arterial grafts, were not evaluated in this study. The consistency of the results obtained using FFR and non-hyperemic methods was evaluated. The agreement of the physiological assessment between FFR and non-hyperemic methods was analyzed for the binary outcome of the mentioned assessments in two ways. First, the frequency of consistent results (both methods indicated myocardial ischemia or lack of ischemia) and discordant results were analyzed, regardless of the type of discordance. Subsequently, the compliance was analyzed, taking into account four groups of patients: The first group included patients in whom both methods indicated ischemia (FFR+|iFR/RFR+); the next were groups in which inconsistent results were obtained, FFR+|iFR/RFR− and FFR−|iFR/RFR+, respectively. The fourth group included vessels for which neither method showed myocardial ischemia (FFR−|iFR/RFR−). Subsequently, factors predisposing to a positive physiological assessment result of borderline stenosis in both study groups were investigated separately for FFR and non-hyperemic evaluation.

Ethics approval (approval number: 1072.6120.257.2022, 16 November 2022) was granted by the institutional ethical board of the Jagiellonian University Medical College for this retrospective registry.

### Statistical Analysis

Categorical variables were presented as numbers and percentages. Continuous variables were expressed as mean, standard deviation (SD), or median with first and third quartiles (Q1–Q3). Differences between groups were compared using Student’s *t*-test for normally distributed variables and the Wilcoxon test for non-normally distributed continuous variables. Categorical variables were compared using Pearson’s chi-squared test. Univariate analyses based on logistic regression for predictors of FFR-positive and iFR/RFR-positive results were presented. Receiver operating characteristic (ROC) curves were created to assess the optimal cutoff values of FFR for predicting iFR/RFR ≤ 0.89 and iFR/RFR for predicting FFR ≤ 0.80. Two-sided *p*-values < 0.05 were considered statistically significant. All calculations were performed using JMP^®^, Version 16.1.0 (SAS Institute Inc., Hong Kong, China).

## 3. Results

Data from 381 patients were collected, with 92 (24.1%) from the younger group and 289 (75.9%) from the older group. In total, 417 vessels were analyzed, including 103 (24.7%) from the younger group and 314 (75.3%) from the older group.

Baseline characteristics showed significant differences between the groups. Older patients were more likely to be women and had a higher prevalence of diabetes mellitus, arterial hypertension, atrial fibrillation, and peripheral vascular disease (Table 1). Additionally, older patients had lower glomerular filtration rates (GFRs). Conversely, the younger group had a higher percentage of active smokers.

Despite the older patients having more risk factors for CAD, their physiological assessment results of borderline coronary stenosis were similar to those of younger patients, as shown in Table 2. In addition, the location of borderline lesions (LAD vs. non-LAD) and the assessment results were comparable between both groups.

Positive FFR assessments were obtained in almost 50% of all evaluations from both age groups. However, the LAD assessments were positive in over 60% of cases for the older patients, but the younger patients had a slightly lower percentage of positive assessments, with no statistically significant difference. Non-LAD lesions were less frequently hemodynamically significant based on FFR, with no significant differences between age groups. Non-hyperemic assessments showed similar FFR results in older patients but slightly less frequent positive iFR/RFR results in younger patients. However, these differences were insignificant (78.6% vs. 81.5%; *p* = 0.52).

In younger patients, cigarette use and type 2 diabetes mellitus increased the risk of a positive FFR result by 3.5 and 2.5 times, respectively. In older patients, male gender and peripheral vascular disease significantly increased the risk of a positive FFR by 2.5 and 2 times, respectively. However, neither smoking nor diabetes mellitus significantly impacted the risk of positive FFR results in the older group (Table 3).

None of the analyzed clinical factors significantly impacted the risk of a positive non-hyperemic assessment in younger patients. In contrast, peripheral vascular disease, insulin-treated diabetes, male gender, and type 2 diabetes diagnosis were identified as risk factors in the older group, increasing the risk of a positive non-hyperemic assessment by around 2–2.5 times. Patient age, analyzed as a continuous variable throughout the entire group of studied patients, did not significantly affect the risk of a positive hyperemic (OR 0.99 (0.98–1.01); *p* = 0.57) or non-hyperemic (OR 1.01 (0.99–1.03); *p* = 0.25) result of the physiological assessment of the coronary circulation. The results of the analyses in the defined subgroups are presented in Table 3. Smoking and sex were the most influential factors for FFR results in younger and older patients, respectively. In older patients, women were less likely to obtain a positive FFR or iFR/RFR result, while sex had no significant effect in younger patients (Table 4). Younger smokers were twice as likely to have a positive FFR result as non-smokers. However, smoking did not affect the iFR/RFR results for younger patients or either assessment for older patients (Table 5).

**Figure 1:**
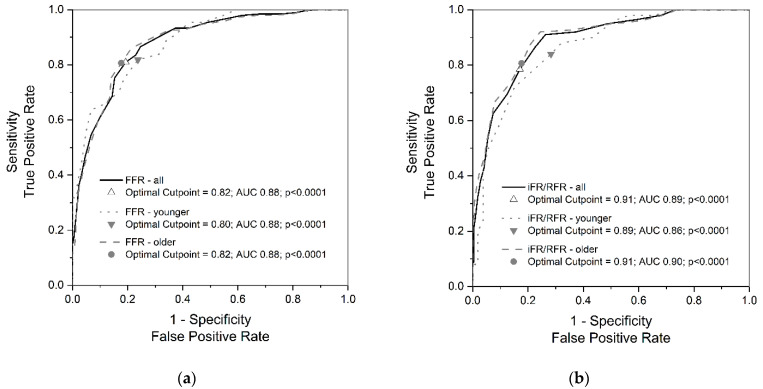
Receiver operating characteristic curves: (**a**) classification accuracy of fractional flow reserve; (**b**) classification accuracy of instantaneous wave-free ratio/resting full-cycle ratio. Abbreviations: AUC, area under curve; FFR, fractional flow reserve; iFR, instantaneous wave-free ratio; RFR, resting full-cycle ratio.

To ascertain the cutoff points of both hyperemic and non-hyperemic evaluations, we plotted ROC curves for FFR (Figure 1a) and for iFR/RFR (Figure 1b). These graphs illustrate that both techniques aptly distinguish the physiological significance of the evaluated alterations, with an AUC for each curve approximating 0.9. Notably, the optimal cutoff for the younger patients aligns seamlessly with the threshold adopted in the publication methodology and conventional clinical protocols. Conversely, the cutoff for the entire cohort and the older patients is marginally elevated compared to commonly accepted tresholds.

## 4. Discussion

The main findings of this study are as follows:

(1) Borderline changes in coronary arteries yield similar physiological assessment results in younger and older patients, with hyperemic assessment producing comparable outcomes. However, the non-hyperemic assessment had slightly more positive results in the older group.

(2) Older patients exhibit more risk factors for CAD than younger patients. However, these factors do not significantly affect the hyperemic evaluation of coronary artery stenoses and only slightly impact non-hyperemic evaluation.

(3) Factors that significantly modify the risk of a positive physiological assessment of borderline coronary stenosis vary with patient age. Smoking is the most important factor in younger patients, while in older patients, the male gender is vital.

Despite multiple risk factors for CAD in older patients, the physiological assessment of borderline coronary stenoses does not yield more positive results than in younger counterparts. This could be due to selection bias and the risk that older patients qualified directly for revascularization without physiological evaluation. Another possibility is the weaker vasodilating response to adenosine in older patients [10], leading to slightly higher hyperemic scores. This phenomenon was not observed in non-hyperemic assessments. The reports suggest that FFR results are slightly higher in patients over 60, causing them to less frequently indicate physiologically significant stenosis compared to non-hyperemic methods [2]. More importantly, our results confirmed that FFR−|iFR/RFR+ discrepancy is more common in older patients, whereas FFR+|iFR/RFR− discrepancy showed an inverse relationship.

As age increases, coronary blood flow during hyperemia decreases. Positron-emission-tomography-based studies have shown more frequent significant changes in the microcirculation in patients over 60 [7]. Consequently, younger patients more often had positive FFR results than non-hyperemic assessment, while older patients had similar frequencies.

In younger patients, smokers have a significantly higher risk of having borderline changes being hemodynamically significant (as assessed by FFR). This risk factor does not affect older patients. Smoking is well-established as one of the most critical risk factors for developing CAD [11], including acute coronary syndromes [12] in younger patients. Miyazaki et al. have confirmed that smoking impacts microcirculatory resistance, lowering smokers’ FFR and CFR values [13]. Their study involved patients with an average age of just over 70 years. Despite higher microcirculatory resistance, FFR was only slightly lower in smokers. These results are consistent with our observations, and, at the same time, they suggest that perhaps the natural course and development of CAD in smokers initially reduces coronary reserve—hence, more frequent positive FFR results in the group of younger patients. Then, with time, CAD causes an increase in microcirculatory resistance, which, in the course of the disease, may slightly increase the FFR value. This hypothesis requires further research but is supported by data showing that short-term smoking reduces the myocardial flow reserve [14]. Additionally, coronary vasomotor abnormalities caused by endothelial damage in smokers may resolve in young smokers [15] after cessation but have a lower chance of resolving in older smokers [16].

In older patients, men are more likely to have hemodynamically significant borderline lesions than women (assessed by FFR), while sex did not affect the risk in younger patients. Previous studies have shown that women tend to have higher FFR results with similar angiographic characteristics, but non-hyperemic scores are comparable for both sexes [17,18,19]. The DEFINE-FLAIR study supports these findings, with women having significantly fewer positive FFR results but similar non-hyperemic assessments [20]. These differences may be due to higher resting coronary blood flow [21], relatively more frequent coronary microcirculation dysfunction [22], larger heart muscle mass in relation to the mass supplied by the assessed artery [23], and smaller coronary artery size [24] in women.

## 5. Limitations

The limitations in this study include the absence of independent coronary angiography analysis, which left the assessment to the operator. Additionally, the non-standardized physiological assessment protocol, based on clinical practice and individual operator decisions, may have influenced results but reflects everyday clinical practice.

## 6. Conclusions

Clinical characteristics and risk factors of patients undergoing physiological assessment of borderline coronary stenosis varied significantly by age. Refining the definition of borderline lesions to include not only angiographic findings but also age, gender, and other clinical factors [25] may improve the ability to identify patients who would benefit from physiological assessment and coronary revascularization. However, this promising approach, supported by the existing literature [26,27], warrants further investigation.

## Figures and Tables

**Table 1:** Baseline clinical characteristics of the study population.

	Group		*p*-Value
	Younger (≤60 y.o.) 92 (24.1%)	Older (>60 y.o.) 289 (75.9%)	
Gender, female, *n* (%)	15 (16.3)	77 (26.6)	0.044
Height, cm, median (Q1–Q3)	172.0 (168.0–177.0)	170.0 (165.0–176.0)	0.030
Weight, kg, median (Q1–Q3)	88.0 (76.0–98.0)	84.0 (73.0–92.0)	0.027
BMI, kg/m^2^, median (Q1–Q3)	29.1 (26.2–32.3)	28.4 (25.3–31.5)	0.23
Diabetes mellitus, *n* (%)	27 (29.4)	127 (43.9)	0.013
Arterial hypertension, *n* (%)	73 (79.4)	258 (89.6)	0.011
Atrial fibrillation, *n* (%)	8 (8.7)	67 (23.3)	0.002
Previous MI, *n* (%)	42 (45.7)	136 (47.1)	0.81
Previous PCI, *n* (%)	40 (43.5)	156 (54.0)	0.08
Previous CABG, *n* (%)	1 (1.1)	13 (4.51)	0.20
PAD, *n* (%)	5 (5.4)	48 (16.7)	0.007
Current smoker, *n* (%)	55 (59.8)	135 (46.7)	0.029
COPD, *n* (%)	4 (4.4)	23 (8.0)	0.24
Previous stroke/TIA, *n* (%)	5 (5.4)	30 (10.4)	0.15
Dyslipidemia, *n* (%)	72 (78.3)	221 (76.5)	0.72
GFR, mL/min/1.73 m^2^, mean (SD)	81.8 (25.9)	74.7 (25.9)	0.025
LVEF, %, median (Q1–Q3)	50.0 (41.0–60.0)	53.0 (40.0–60.0)	0.75
Radial access, *n* (%)	78 (84.8)	235 (81.3)	0.45

Abbreviations: BMI, body mass index; CABG, coronary artery bypass grafting; COPD, chronic obstructive pulmonary disease; GFR, glomerular filtration rate; LVEF, left ventricle ejection fraction; MI, myocardial infarct; PAD, peripheral arterial disease; PCI, percutaneous coronary intervention; TIA, transient ischemic attack.

**Table 2:** The results of vessel assessment in the study groups (per vessel).

	Group		*p*-Value
	Younger (≤ 60 y.o.) 103 (24.7%)	Older (>60 y.o.) 314 (75.3%)	
Vessel assessed			
LAD	64 (62.1%)	185 (58.9%)	0.56
non-LAD	39 (37.9%)	129 (41.1%)	
All vessels			
FFR ≤ 0.80, *n* (%)	50 (48.5%)	150 (47.8%)	0.89
FFR, median (Q1–Q3)	0.81 (0.75–0.87)	0.815 (0.76–0.88)	0.52
iFR/RFR ≤ 0.89, *n* (%)	44 (42.7%)	150 (47.8%)	0.37
iFR/RFR, median (Q1–Q3)	0.90 (0.87–0.94)	0.90 (0.85–0.94)	0.50
LAD			
FFR ≤ 0.80, *n* (%)	35 (54.7%)	114 (61.6%)	0.33
FFR, median (Q1–Q3)	0.80 (0.75–0.85)	0.78 (0.73–0.84)	0.43
iFR/RFR ≤ 0.89, *n* (%)	34 (53.1%)	111 (60.0%)	0.34
iFR/RFR, median (Q1–Q3)	0.89 (0.8625–0.92)	0.88 (0.83–0.92)	0.16
Non-LAD			
FFR ≤ 0.80, *n* (%)	15 (38.5%)	36 (27.9%)	0.21
FFR, median (Q1–Q3)	0.83 (0.76–0.90)	0.86 (0.80–0.91)	0.16
iFR/RFR ≤ 0.89, *n* (%)	10 (25.6%)	39 (30.2%)	0.58
iFR/RFR, median (Q1–Q3)	0.93 (0.88–0.97)	0.94 (0.88–0.97)	0.89
Concordance-general			
concordant	81 (78.6%)	256 (81.5%)	0.52
discordant	22 (21.4%)	58 (18.5%)	
FFR−|iFR/RFR−	45 (43.7%)	135 (43.0%)	0.60
FFR−|iFR/RFR+	8 (7.8%)	29 (9.2%)	
FFR+|iFR/RFR−	14 (13.6%)	29 (9.2%)	
FFR+|iFR/RFR+	36 (35.0%)	121 (38.5%)	

Abbreviations: FFR, fractional flow reserve; iFR, instantaneous wave-free ratio; LAD, left anterior descending artery; RFR, resting full-cycle ratio.

**Table 3:** Univariate analysis for predictors of positive results of coronary stenosis assessment.

	Crude OR Younger Group (95% Confidence Interval)	*p*-Value	Crude OR Older Group (95% Confidence Interval)	*p*-Value
Predictors of FFR+				
Gender, male	1.35 (0.50–3.76)	0.54	2.45 (1.46–4.12)	0.001
DM, yes	2.62 (1.13–6.05)	0.015	1.44 (0.92–2.26)	0.11
PAD, yes	5.78 (0.65–51.31)	0.12	1.86 (1.05–3.30)	0.034
Smoking, yes	3.49 (1.42–8.62)	0.007	1.07 (0.69–1.67)	0.75
Age (continuous, per 1 year)	0.97 (0.90–1.04)	0.35	0.99 (0.96–1.03)	0.73
Predictors of iFR/RFR+				
Gender, male	1.35 (0.48–3.77)	0.57	1.74 (1.05–2.88)	0.032
DM, yes	0.66 (0.29–1.51)	0.32	1.69 (1.08–2.65)	0.023
PAD, yes	1.37 (0.26–7.11)	0.71	2.41 (1.34–4.34)	0.003
DM on insulin, yes	1.26 (0.28–5.65)	0.76	2.25 (1.12–4.55)	0.023
Age (continuous, per 1 year)	0.93 (0.87–1.00)	0.056	1.03 (0.99–1.06)	0.08

Abbreviations: DM, diabetes mellitus; FFR, fractional flow reserve; iFR, instantaneous wave-free ratio; OR, odds ratio; PAD, peripheral arterial disease; RFR, resting full-cycle ratio.

**Table 4:** The results of vessel assessment according to the gender (per vessel).

	Male Group (*n* = 311)	Female Group (*n* = 106)	*p*-Value
All vessels-younger group			
FFR ≤ 0.80, *n* (%)	42 (50.0)	8 (42.1)	0.53
FFR, median (Q1–Q3)	0.805 (0.7525–0.86)	0.86 (0.74–0.9)	0.44
iFR/RFR ≤ 0.89, *n* (%)	37 (44.1)	7 (36.8)	0.57
iFR/RFR, median (Q1–Q3)	0.90 (0.87–0.94)	0.93 (0.86–0.97)	0.42
All vessels-older group			
FFR ≤ 0.80, *n* (%)	122 (53.7)	28 (32.2)	0.001
FFR, median (Q1–Q3)	0.8 (0.75–0.86)	0.85 (0.78–0.91)	<0.001
iFR/RFR ≤ 0.89, *n* (%)	117 (51.5)	33 (37.9)	0.031
iFR/RFR, median (Q1–Q3)	0.89 (0.84–0.94)	0.92 (0.87–0.95)	0.06

Abbreviations: FFR, fractional flow reserve; iFR, instantaneous wave-free ratio; RFR, resting full-cycle ratio.

**Table 5:** Results of vessel assessment according to smoking status (per vessel).

	Smoking Group (*n* = 223)	Non-Smoking Group (*n* = 194)	*p*-Value
All vessels-younger group			
FFR ≤ 0.80, *n* (%)	41 (57.8)	9 (28.1)	0.005
FFR, median (Q1–Q3)	0.79 (0.75–0.86)	0.845 (0.8–0.9)	0.006
iFR/RFR ≤ 0.89, *n* (%)	34 (47.9)	10 (31.3)	0.11
iFR/RFR, median (Q1–Q3)	0.90 (0.86–0.94)	0.915 (0.89–0.94)	0.15
All vessels-older group			
FFR ≤ 0.80, *n* (%)	74 (48.7)	76 (46.9)	0.75
FFR, median (Q1–Q3)	0.81 (0.76–0.87)	0.83 (0.76–0.89)	0.17
iFR/RFR ≤ 0.89, *n* (%)	76 (50.0)	74 (45.7)	0.44
iFR/RFR, median (Q1–Q3)	0.895 (0.84–0.94)	0.90 (0.8575–0.94)	0.76

Abbreviations: FFR, fractional flow reserve; iFR, instantaneous wave-free ratio; RFR, resting full-cycle ratio.

## Data Availability

The data presented in this study are available on request from the corresponding author.
